# Optimization for Service Routes of Pallet Service Center Based on the Pallet Pool Mode

**DOI:** 10.1155/2016/5691735

**Published:** 2016-07-26

**Authors:** Kang Zhou, Shiwei He, Rui Song

**Affiliations:** ^1^School of Traffic and Transportation, Beijing Jiaotong University, Beijing 100044, China; ^2^MOE Key Laboratory for Urban Transportation Complex Systems Theory and Technology, Beijing Jiaotong University, Beijing 100044, China

## Abstract

Service routes optimization (SRO) of pallet service center should meet customers' demand firstly and then, through the reasonable method of lines organization, realize the shortest path of vehicle driving. The routes optimization of pallet service center is similar to the distribution problems of vehicle routing problem (VRP) and Chinese postman problem (CPP), but it has its own characteristics. Based on the relevant research results, the conditions of determining the number of vehicles, the one way of the route, the constraints of loading, and time windows are fully considered, and a chance constrained programming model with stochastic constraints is constructed taking the shortest path of all vehicles for a delivering (recycling) operation as an objective. For the characteristics of the model, a hybrid intelligent algorithm including stochastic simulation, neural network, and immune clonal algorithm is designed to solve the model. Finally, the validity and rationality of the optimization model and algorithm are verified by the case.

## 1. Introduction

 In the pallet pool mode, the usage rate of pallet can be improved and logistics cost can be reduced by duty-cycle operation. However, this will increase the liquidity of the pallets. After pallet service center is determined, the pallets are circulated in the circulation of service center, each demand point and freight station by the scheduling and coordination of service center and according to the actual supply and demand situation.

The purpose of pallet service route optimization is to determine the optimal service path, so that the vehicle can provide services (delivery and recovery operation) for each demand point efficiently. This is a problem of solving the optimal routes for multipoints. Now, a few researches for the route optimization of pallet service center are reported in the literature. Through analyzing the purpose of pallet service route optimization, we find that the pallet service route optimization is similar to logistics distribution vehicle routing optimization (VRP), so we can draw lessons from the relevant theory research of VRP.

Vehicle routing optimization problem is defined as follows: in the case of warehouse and when the customer's position is fixed, the distance, the demand, and other information are all known. Take the customer's various demand as the restraint condition, and seek the best plan to achieve the shortest path (the province with the highest expense, the least time, etc.) [1]. Due to the complexity of the actual demand, the vehicle routing problem with the related constraints based on the basic vehicle routing optimization problem is generated. Capacitated vehicle routing problem (CVRP) takes into account the vehicle loading capacity; each vehicle can only be loaded with a certain amount of goods, and vehicles' capacity must be increased when the goods exceed the vehicle loading capacity. CVRP is the most common vehicle routing problem. Vehicle routing problem with time windows (VRPTW) mainly researches the service time according to the requirement of customers; service time is limited by customer; the vehicle can only arrive at destination within the specified time; the service is not accepted or the service is punished with penalty at other times. The vehicle routing problem with time windows is a common problem in reality, especially for urban customers or when there are many random transportation processes [2]. Vehicle routing problem with pickups and deliveries (VRPPD) considers the demand of delivery and recovery for each customer; delivery and recovery services need to be completed at the same time for one customer; this can save the vehicle transportation cost for the logistics enterprises. It is a subproblem with economic efficiency and operating efficiency of the vehicle routing optimization problem, but it does not consider the time constraint [3]. Psaraftis [4, 5] presented a dynamic vehicle routing problem. Since then, many scholars have done a lot of research in this field. Larsen [6] studied the method of dynamic degree feature index of DVRP. Brotcorne et al. [7] studied the dynamic scheduling problem of emergency vehicles, which is mainly about the emergence of new customers online. Taniguchi and Shimamoto [8] and Fleischmann et al. [9] studied the problem of real-time traffic information of DVRP. Melachrinoudis et al. [10] considered the impact of the special environment of medical service for the DVRP and constructed the corresponding model. Khouadjia et al. [11] and Ferrucci et al. [12], respectively, studied DVRP with dynamic customer, and the corresponding solving strategy was put forward. Albareda-Sambola et al. [13] verified the feasibility of dividing DVRP by time slots. Ghannadpour et al. [14] studied DVRP of multiobjective.

In addition, the delivery and recovery operation of pallet service center is similar to the operation of sending and receiving mails, so the route optimization can also learn from the Chinese postman problem. The Chinese postman problem as an important research field of operational research was first proposed by Professor Guan [15]; the problem is described as follows: the postman executes delivery task from the post office; he is required to select one of the shortest routes and must pass through the streets at least once and then return to the post office. Subsequently, the Chinese postman problem drew the attention of other scholars around the world [16]. Wang [17] for the first time put forward the “multipostman problem”; several mathematical models of the Chinese postman problem for multipostman were constructed; an effective algorithm for limiting conditions and the computational complexity of the Chinese postman problem for multipostman under normal circumstances were given. Rong et al. [18] put forward the model of multistage decision process conversion algorithm, so as to solve the dynamic programming model of the Chinese postman problem for multipostman. Zhu [19] proposed a single center Royd algorithm of the Chinese postman problem for multipostman and iterative diagram of computer program. “Multipostman problem” is the extension of the Chinese postman problem; it can be described as follows: a post office has *N* (*N* ≥ 2) postmen; they were set out at the same time to send and receive messages of each street and finally returned to the post office. It is required that the total delivery route is the shortest by the reasonable allocation of the delivery route for each postman.

## 2. Problem Description and Model Construction

For any pallet service area, the supply points, demand points, and service centers have fixed distribution. When providing supply and demand service (delivery and recovery operation), it is necessary that the distribution vehicle traverses the demand points along the optimal route in order to achieve high efficiency of service operation with the minimum cost. This is consistent with the Chinese postman problem; it can be described using a mathematical language: if the road is expressed with edge (*u*, *v*), the length of the road is expressed with edge weight *w*(*u*, *v*), and the pallet service center, freight station, and pallet demand enterprise are all expressed with point, so a pallet service area constitutes an undirected graph connected with edge weight. Routes optimization of pallet service center can be described with the language of graph theory, that is, how to find a loop *C* in an undirected graph connected with edge weight which passes each edge at least once, where the length of *C* is the shortest and *C* is the best service route in the service area.

Different from the logistics distribution, pallet service includes delivery and recovery. Generally speaking, the pallets used by customers cannot be provided to other customers directly; they should be recovered to the service center and be inspected, cleaned, and repaired. Finally, they are sent to the customer. Therefore, in order to make the total service routes the shortest, in every service work, we should first determine the type and amount of the pallets and vehicles according to pallet demand and recovery information and then find the optimal route for each vehicle.

### 2.1. Optimization Method of Service Route

Based on the analysis of the previous section, we use solution ideas of the Chinese postman problem and draw the vehicle routing problem to solve the route optimization problem of pallet service center. Before using the Chinese postman problem to optimize the route, it is necessary to describe concepts of the Chinese postman problem.

**Definition 1 .**

The trace along each edge of the graph *G* (*G* = (*V*, *E*), where *V* is the set of nodes and *E* is the set of sections) is called Euler trace of *G*. The closed trajectory of Euler is called Euler loop or *E* loop, and the graph including Eulerian circuit is called Euler graph.

Euler loop is the graph that starts from a vertex and goes back to the starting point along each edge that is not repeated.

In the Chinese postman problem, there are (*n* − 1)! ways of arranging for *n* delivery points without the starting point; each arrangement is delivery route planning. With the increase of *n*, the amount of computation shows exponential growth, and the time spent by the enumeration method is difficult to bear.

Based on the related literatures, the Chinese postman problem can be converted to 0-1 programming.

First, we determine whether each edge is included in the delivery line.

*x*_*u*,*v*_  (*u* ≠ *v*) is 0-1 integer variable; *x*_*u*,*v*_ = 1 indicates that the route is from *u* to *v*, that is, (*u*, *v*) in the route; *x*_*u*,*v*_ = 0 means (*u*, *v*) is not in the route.

The objective function is as follows:

(1) min z=∑u=1n ∑v=1ncu,vxu,v,

where *c*_*u*,*v*_ is the cost of vehicle running in the route (*u*, *v*).

The constraint of visit frequency for demand points is as follows:

(1) Starting from the delivery point *u* once and only once (to the other delivery points), it is expressed as
  (2) $$\begin{array}{c} \overset{n}{\underset{v=1}{\sum }}x_{u,v}=1,\;u=1,2,\ldots ,n,\;\;v\neq u. \end{array}$$
(2) From a delivery point to *v* once and only once, it is expressed as
  (3) $$\begin{array}{c} \overset{n}{\underset{u=1}{\sum }}x_{u,v}=1,\;v=1,2,\ldots ,n,\;\;u\neq v. \end{array}$$

The model established above is similar to the model of assignment problem; it is only a necessary condition for the Chinese postman problem, but not sufficient condition.

**Figure 1:**
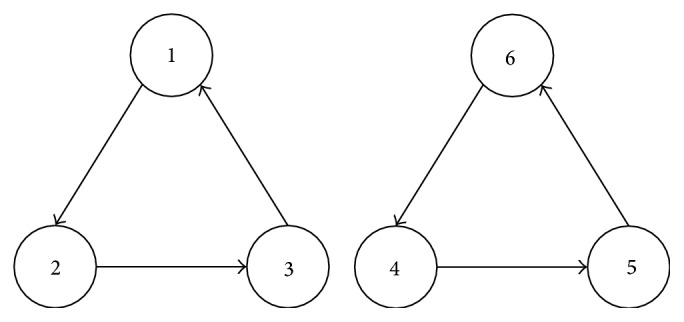
Disconnected subcircuits.

For example, there are six points connected by the routes (as shown in Figure 1); they meet the above two constraints, but there are two unreasonable subcircuits, so they cannot constitute the whole circuit.

For this situation, we need to consider increasing the sufficient conditions to avoid the subcircuit.

Add a variable *k*_*u*_, *u* = 1,2,…, *n* (its size can be taken as an integer, e.g., the delivery point *u* = 2, which is achieved by the vehicle from start point).

An additional constraint is as follows:

(4) ku−kv+nxu,v≤n−1,u=1,…,n,  v=2,…,n,  u≠v.

We prove the constraint as follows:

(1) Any line that contains the subcircuit cannot meet the constraint (no matter what the value of *k*_*u*_ is).
(2) The whole circuit without the subcircuit can meet the constraint (as long as *k*_*u*_ takes the proper value).

We use apagoge to prove (1), if there are subcircuits, at least two. Then, at least one subcircuit does not include the starting point 1, as shown in Figure 1; then, there will be

(5) k4−k5+n≤n−1,k5−k4+n≤n−1,k6−k4+n≤n−1.

Add these three inequalities and get *n* ≤ *n* − 1; this is obviously incorrect, so the assumption cannot be established. For the whole circuit, because of *v* ≥ 2 in the additional constraints, it does not contain the starting point 1, so it will not be contradictory.

For the whole circuit, as long as *k*_*u*_ takes the appropriate value, it can meet the constraints:

(1) For the edge of the whole circuit, *x*_*u*,*v*_ = 1, *k*_*u*_ can take the order number of access point *u*; then there will be *k*_*u*_ − *k*_*v*_ = −1, and constraint condition *k*_*u*_ − *k*_*v*_ + *nx*_*u*,*v*_ ≤ *n* − 1 becomes −1 + *n* ≤ *n* − 1; this is inevitable.
(2) For the edge of nonwhole circuit, because of *x*_*u*,*v*_ = 0, the constraint condition *k*_*u*_ − *k*_*v*_ + *nx*_*u*,*v*_ ≤ *n* − 1 becomes −1 ≤ *n* − 1; this is inevitable.

In summary, the constraint condition only restricts the subcircuit and does not have other effects. So, the Chinese postman problem is transformed into a mixed integer linear programming problem.

### 2.2. Service Route Problem Formulation Based on Multipostman Problem

#### 2.2.1. Assumptions for Modeling

Before modeling, the following preconditions and assumptions are given:

1. The influence of pallet type for delivery and recovery is ignored.
2. Each pallet service center is only responsible for pallet delivery and recovery within its own jurisdiction range.
3. Each vehicle has only one transport route; the start and end points of each route are the same pallet service center.
4. Each vehicle can provide service for more than one demand point, but each demand point can only be provided by one vehicle. For the demand of demand point more than a single vehicle loading capacity, we should determine the number of vehicles required to load the pallets and only take the amount of pallets which is less than one vehicle as the optimization target.
5. Each demand point is put forward to the corresponding requirements for service time; service vehicles must arrive at the point within the time limits specified by the time window; the vehicle in each demand point of delivery time is random.
6. In the decision period, the amount of pallets waiting to be delivered and recovered is known and is random.

#### 2.2.2. Parameters Definition

*(1) Sets. A* is the set of service centers, *A* = {*i*∣*i* = 1,2,…, *n*}; *V* is the set of demand points (the pallet demand enterprises, freight stations, and terminal companies are all viewed as demand points), *V* = {*v*∣*v* = 1,2,…, *m*}; *Q* is the number of vehicles, *Q* = {*q*∣*q* = 1,2,…, *k*}, where *q* is one of the elements, *q* ∈ *Q*; *S* is the number of steps for the vehicle (demand point of arriving), *s* ∈ *S*.

*(2) Parameters and Decision Variables.* The number of demand points serviced by vehicle *q* is *m*_*q*_, and ∑_*q*=1_^*k*^ *m*_*q*_ = *m*, so *S* = {*s*∣*s* = 1,2,…, *m*_*q*_}; *P*_*v*_ is the amount of pallets that need to be distributed to the demand point *v*; *Q*_*v*_ is the amount of pallets that need to be recycled from the demand point *v*; *W*_*s*_^*q*^ is the amount of loading after the vehicle *q* runs *s* steps; *d*_*i*_ is the amount of pallets readied for distribution of pallet service center *i* in the decision period; *f*_*i*_ is the amount of pallets readied for recovering of pallet service center *i* in the decision period; *r*_*q*_ is the maximum loading of the vehicle *q*; *l*_*v*_ is the distance from the service center to the demand points; *C*_*u*,*v*_ is the shortest path between *u* and *v*; *T*_*v*_^*q*^ is the time of vehicle *q* arriving at the demand point *v*; *t*_0_^*q*^ is the departure time of vehicle *q* from the service center; *t*_*u*,*v*_^*q*^ is the theoretical traveling time of vehicle *q* from the point *u* to *v*; *t*_*v*_^*q*^ is the service time of the vehicle *q* at the point *v*; [*a*_*v*_, *b*_*v*_] is the time window of servicing required by point *v*; *a*_*v*_ and *b*_*v*_ denote the start and end time, respectively.

*L*_*q*_ represents the distance of vehicle *q* traveling from the service center to the first demand point and from the last demand point to the service center; *L*_*q*_^*x*^ represents the distance between the first demand point and the last demand point of vehicle *q* that arrived; *X*_*s*,*v*_^*q*^ is the 0-1 decision variable; it represents the vehicle *q* at the step *s* reaching the demand point *v*; subscript *s* represents the number of steps that vehicle *q* passed; and *v* indicates the arriving demand point. *X*_*s*,*v*_^*q*^ = 1 expresses that the loading and unloading points of vehicle *q* that arrived at the step *s* are demand point *v*; otherwise, *X*_*s*,*v*_^*q*^ = 0.

#### 2.2.3. Model Building

First, it is necessary to use the fewest vehicles in the process of pallets delivering and recovering; the number of vehicles is determined according to the amount of pallets waiting for distribution and recycling. The formula is *Q* = [max⁡(*d*_*i*_, *f*_*i*_)/*r*_*q*_], and [·] expresses the rounding operation.

For example, the total number of pallets waiting for distribution is *d*_*i*_ = 1760, and the total number of pallets waiting for recycling from the demand points is *f*_*i*_ = 1400; each vehicle can accommodate up to 355 pieces; the minimum number of vehicles required is *Q* = [max(1760,1400)/355] = 5.

The number of service routes is determined by the above method. Vehicles start from the service center, passing some demand points to load and unload, and finally come back to the service center.

The demand points within the service area are considered as a whole; the number of service routes *Q* is determined. The demand points of the vehicles passing are unknown; by reasonably arranging the transport routes of the vehicle, the *Q* vehicles are determined corresponding to the routes. Under the premise of meeting the transportation capacity, take the total mileage of the *Q* routes minimum as the objective. The problem is equivalent to the multipostman problem with constraints.

Based on the above analysis, the objective function and the constraint conditions of service routes optimization are given.

*(1) Objective Function.* Take the total mileage minimum of all vehicles completing a delivery and recovery operation in the decision period as an objective; the function is

(6) min z=∑q=1kLq+Lqx,

where *L*_*q*_ = ∑_*v*=1_^*m*^ *l*_*v*_ · (*X*_1,*v*_^*q*^ + *X*_*m*_*q*_,*v*_^*q*^), ∀*q* ∈ *Q*, and *L*_*q*_^*x*^ = ∑_*s*=1_^*m*_*q*_−1^∑_*u*=1_^*m*^∑_*v*=1_^*m*^ *C*_*u*,*v*_ · *X*_*s*,*u*_^*q*^ · *X*_*s*+1,*v*_^*q*^, ∀*q* ∈ *Q*.

*(2) Constraint Conditions.* (1) For any vehicle in any one step arriving at one and only one demand point,

(7) $$\begin{array}{c} \overset{m}{\underset{v=1}{\sum }}X_{s,v}^{q}=1,\;\forall q\in Q,\;\;s\in S. \end{array}$$

(2) For any demand point, there must be a vehicle arriving once and only once:

(8) $$\begin{array}{c} \overset{k}{\underset{q=1}{\sum }}\overset{m_{q}}{\underset{s=1}{\sum }}X_{s,v}^{q}=1,\;\forall v\in V. \end{array}$$

(3) Loading constraint: in the process of delivering and recycling for pallets, the loading quantity exhibits a dynamic change in transport process; considering the uncertainty of damage and demand in the transport process, the stochastic demand is added for pallets distribution, in order to deal with the uncertainty of demand. Take the vehicle *q* as an example; the loading quantity at the time of departure is

(9) $$\begin{array}{c} W_{0}^{q}=\overset{m}{\underset{v=1}{\sum }}\left[\;\left(\;P_{v}+\gamma_{v}\;\right)\cdot \overset{m_{q}}{\underset{s=1}{\sum }}X_{s,v}^{q}\;\right],\;\forall q\in Q, \end{array}$$

where *γ*_*v*_ is random demand.

After the operation of loading and unloading at the first demand point, the loading quantity is

(10) $$\begin{array}{c} W_{1}^{q}=W_{0}^{q}-\overset{m}{\underset{v=1}{\sum }}\left(\;P_{v}+\gamma_{v}-Q_{v}\;\right)X_{1,v}^{q},\;\forall q\in Q. \end{array}$$

In the process of delivering and recycling, the recursive formula of the loading quantity is

(11) Wsq=Ws−1q−∑v=1mPv+γv−Qv·Xs,vq,∀q∈Q,  s∈S,  s≥1.

The constraint of loading quantity is

(12) $$\begin{array}{c} W_{s}^{q}\leq r_{q},\;\forall q\in Q,\;\;s\in S. \end{array}$$

(4) Time constraints: the departure time of vehicle *q* from service center is

(13) $$\begin{array}{c} T_{0}^{q}=t_{0}^{q},\;\forall q\in Q. \end{array}$$

The time of arriving at the first demand point is

(14) $$\begin{array}{c} T_{1}^{q}=t_{0}^{q}+\overset{m}{\underset{v=1}{\sum }}t_{0,v}^{q}\cdot X_{1,v}^{q},\;\forall q\in Q. \end{array}$$

In the process of delivering and recycling, the recursive formula of vehicle arriving at each demand point is

(15) Tvq=t0q+∑v=1mtv−1,vq·Xs,vq+∑v=1mtv−1q·Xs−1,v−1q,∀q∈Q,  s∈S.

According to the above analysis, *T*_*v*_^*q*^ should meet the following conditions:

(16) av≤Tvq≤bv,∀q∈Q,  v∈V,or  Tvq∈av,bv,∀q∈Q,  v∈V.

Due to the uncertainty of vehicles in the transport process, they cannot arrive at the point in the required time window completely; there will be more or less some deviations, this is expressed as follows:

(17) Tvq∈av−dv−,bv+dv+,∀q∈Q,  v∈V,or  av−dv−≤Tvq≤bv+dv+,∀q∈Q,  v∈V,

where *d*_*v*_^+^ and *d*_*v*_^−^ are time variables of random arrival, *d*_*v*_^+^ indicates a positive deviation from the target value *b*_*v*_ of vehicle arriving at the demand point *v*, and *d*_*v*_^−^ indicates a negative deviation from the target value *a*_*v*_ of vehicle arriving at the demand point *v*.

Therefore, the 0-1 programming model of service route is as follows:

(18) min⁡ z=∑q=1kLq+Lqx,s.t. ∑v=1mXs,vq=1,∀q∈Q,  s∈S, ∑q=1k∑s=1mqXs,vq=1,∀v∈V, W0q=∑v=1mPv+γv·∑s=1mqXs,vq,∀q∈Q, W1q=W0q−∑v=1mPv+γv−QvX1,vq,∀q∈Q, Wsq=Ws−1q−∑v=1mPv+γv−Qv·Xs,vq,∀q∈Q,  s∈S,  s≥1, Wsq≤rq,∀q∈Q,  s∈S, T0q=t0q,∀q∈Q, T1q=t0q+∑v=1mt0,vq·X1,vq,∀q∈Q, Tvq=t0q+∑v=1mtv−1,vq·Xs,vq+∑v=1mtv−1q·Xs−1,v−1q,∀q∈Q,  s∈S, Tvq∈av−dv−,bv+dv+,∀q∈Q,  v∈V, Xs,vq=0  or  1,∀q∈Q,  s∈S,  v∈V.

## 3. Solution to the Model

The traditional method for solving the constrained programming model is by converting the opportunity constraint into its equivalent form and then solving the equivalent model. Combining the theory of stochastic constraints, constraints (12) and (17) can be transformed into the chance constraints [20].

Due to the random demand, for the vehicle, the initial loading quantity containing the random demand should be no more than the maximum loading capacity, and each vehicle needs to meet the loading constraints at the specified confidence level *α*. In addition, each demand point is served by a confidence level *β*_*v*_ within its specified time window [*a*_*v*_, *b*_*v*_]. To meet the above conditions, the following chance constraints were given:

(19) PrWsq≤rq≥α,∀q∈Q,  s∈S,PrTvq−bv≤dv+≥βv,∀q∈Q,  v∈V,Prav−Tvq≤dv−≥βv,∀q∈Q,  v∈V.

Because the deterministic processing of stochastic constraint programming is only suitable for special cases, the hybrid intelligent algorithm based on stochastic simulation, neural network, and immune clonal algorithm is used to solve this model. Compared with genetic algorithm, immune clonal algorithm can promote or inhibit the generation of antibodies; it embodies the self-regulation function of the immune system and ensures the diversity of individual.

The solving steps of hybrid intelligent algorithm are as follows.

**Step 1 .**

Employ stochastic simulation technique to generate input-output data for the uncertain function.

**Step 2 .**

Train a neural network to approximate the uncertain function according to the input and output data generated.

**Step 3 .**

Initialize the antibody population and use the trained neural network to test the feasibility of antibody.

**Step 4 .**

Use immune gene operation to update antibody and examine the feasibility of offspring using the trained neural network.

**Step 5 .**

Use the trained neural network to calculate the target values of all antibodies.

**Step 6 .**

The fitness of each antibody is calculated according to the target values.

**Step 7 .**

Select antibodies by clonal selection.

**Step 8 .**

Repeat Step 4 to Step 7 until the end condition is met.

**Step 9 .**

Output optimal solution.

### 3.1. Antibody Description

**Figure 2:**
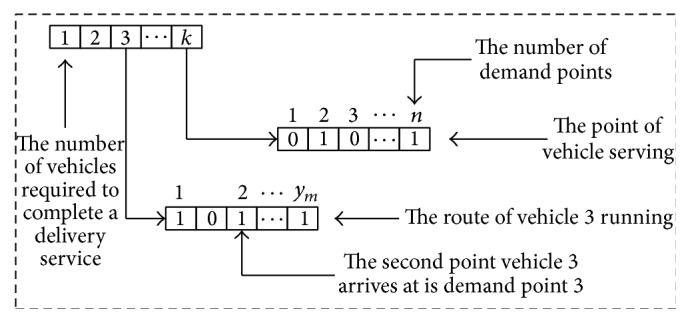
Schematic plot of antibody coding.

*X*_*s*,*v*_^*q*^ is decision variable of service route optimization. It signifies vehicle *q* arriving at the demand point *v* at the *s* step. When using hybrid intelligent algorithm to solve the optimization model of pallet service routes, we make the alternative routes as encoding of solution, as shown in Figure 2.

Use antibody **v** = (**x**, **y**, **t**) to represent a decision-making process, where the gene **x** = (*x*_1_, *x*_2_, *x*_3_,…, *x*_*n*_) expresses the demand points and **y** = (*y*_1_, *y*_2_, *y*_3_,…, *y*_*m*−1_) represents the number of steps for the vehicle running in the service process, and *y*_0_ ≡ 0 ≤ *y*_1_ ≤ *y*_2_ ≤ *y*_3_ ≤ ⋯≤*y*_*m*−1_ ≤ *n* ≡ *y*_*m*_, where 0 represents service center, and t = (*t*_1_, *t*_2_, *t*_3_,…, *t*_*k*_), where *t*_*q*_ represents starting time for the vehicle *q* at service center, *q* = 1,2,…, *k*. For the vehicle *q* (the number of points that need to be served is *v*), if *y*_*m*_ = *y*_*m*−1_, this indicates that the vehicle *q* is not running; if *y*_*m*_ > *y*_*m*−1_, this indicates that the vehicle has been set off. And the time of departure from the service center is *t*_*q*_. Delivery route of vehicle *q* can be expressed as 0 → *x*_*y*_1_,1_^*q*^ → *x*_*y*_2_,2_^*q*^ → ⋯→*x*_*y*_*m*−1_,*v*_^*q*^ → 0.

### 3.2. Initialization of Antibody Population

First, for the gene **x**, we define *x*_*i*_ = *i* and give a sequence {*x*_1_, *x*_2_, *x*_3_,…, *x*_*n*_}, *i* = 1,2, 3,…, *n*, and then repeat the following procedure from *j* to *n*: a random position *n*′ is generated between *j* and *n*, and the value of *x*_*j*_ and *x*_*n*′_ is exchanged, in order to ensure {*x*_1_, *x*_2_, *x*_3_,…, *x*_*n*_} is a rearrangement for {1,2, 3,…, *n*}. So, we get the gene **x** = {*x*_1_, *x*_2_, *x*_3_,…, *x*_*n*_}.

Second, for each *i*  (1 ≤ *i* ≤ *m* − 1), take *y*_*i*_ as random numbers (0 ≤ *y*_*i*_ ≤ *n*); then, they are rearranged in the order of small to large; the sequence {*y*_1_, *y*_2_, *y*_3_,…, *y*_*m*−1_} is generated; that is, **y** = (*y*_1_, *y*_2_, *y*_3_,…, *y*_*m*−1_).

Finally, for *i* = 1,2, 3,…, *k*, let *t*_*i*_ be a random number of departure times [*a*, *b*]; then, **t** = (*t*_1_, *t*_2_, *t*_3_,…, *t*_*k*_).

If it is proved that the antibody **v** = (**x**, **y**, **t**) is feasible, it is acceptable; otherwise, repeat the above process until a viable antibody is obtained.

### 3.3. Clone Operation

Perform clone operation for antibody populations *V*; update *V* to *V*′:

(20) V′TcV=v1′x,y,t,v2′x,y,t,…,vk′x,y,tT,

where **v**_*i*_′(**x**, **y**, **t**) = *T*_*c*_(**v**_*i*_(**x**, **y**, **t**)) = *I*_*i*_ × **v**_*i*_(**x**, **y**, **t**), *i* = 1,2,…, *k*, and *I*_*i*_ is the *q*_*i*_-dimensional row vector where elements are 1, namely, *q*_*i*_ clone of antibody **v**_*i*_(**x**, **y**, **t**). For the single antibody **v**_*i*_(**x**, **y**, **t**), the clone size should be properly adjusted according to the affinity between antibodies and the affinity between antibody and antigen. According to formula (21), the clone size is calculated:

(21) qix,y,t=ceilnc·fvi′x,y,t∑j=1kfvix,y,t·φi,i=1,2,…,k,

where ceil(·) is the function of rounding up and *n*_*c*_ > *k* is the setting value associated with the clone size. *f*(**v**_*i*_(**x**, **y**, **t**)) is affinity between antibody **v**_*i*_(**x**, **y**, **t**) and antigen (fitness); *φ*_*i*_ is the affinity between antibody *i* and other antibodies; the value can be calculated according to the following formula:

(22) $$\begin{array}{c} \varphi_{i}=\min\left\{\;\exp\left(\;\left\|\;v_{i}-v_{j}\;\right\|\;\right)\;\right\},\;i\neq j,\;\;i,j=1,2,\ldots ,k, \end{array}$$

where ‖·‖ is Euclidean distance after normalization; that is, 0 ≤ ‖·‖ ≤ 1. Obviously, the lower the antibody affinity and the higher the similarity degree, the stronger the inhibition among antibodies and the smaller the value of *φ*_*i*_.

### 3.4. Immune Gene Operation

*(1) Crossover Operation.* Crossover operation is executed for any antibodies **v**_1_ and **v**_2_, where **v**_1_ = (**x**_1_, **y**_1_, **t**_1_), **v**_2_ = (**x**_2_, **y**_2_, **t**_2_).

First, a random number *c* is generated in the open interval (0,1), and **t**_1_′ = *c* · **t**_1_ + (1 − *c*) · **t**_2_, **t**_2_′ = (1 − *c*) · **t**_1_ + *c* · **t**_2_. Two subgenerations **v**_1_′ and **v**_2_′ were obtained by crossover operation, where **v**_1_′ = (**x**_1_, **y**_2_, **t**_1_′), **v**_2_′ = (**x**_2_, **y**_1_, **t**_2_′).

*(2) Mutation Operation.* For the gene **x** of antibody **v** = (**x**, **y**, **t**), two mutation positions of *n*_1_ and *n*_2_ are randomly generated between 1 and *n*, and the sequence {*x*_*n*_1__, *x*_*n*_1_+1_, *x*_*n*_1_+2_,…, *x*_*n*_2__} is rearranged to obtain a new sequence {*x*_*n*_1__′, *x*_*n*_1_+1_′, *x*_*n*_1_+2_′,…, *x*_*n*_2__′}, so the new gene is

(23) x′=x1,x2,…,xn1−1,xn1′,xn1+1′,xn1+2′,…,xn2′,xn2+1,xn2+2,…,xn.

Similarly, for the gene **y**, two mutation positions of *n*_1_ and *n*_2_ are randomly generated between 1 and *m* − 1; *y*_*i*_ is a random number between 0 and *n*, *i* = *n*_1_, *n*_1_ + 1, *n*_1_ + 2,…, *n*_2_. The sequence {*y*_1_, *y*_2_,…, *y*_*n*_1_−1_, *y*_*n*_1__′, *y*_*n*_1_+1_′,…, *y*_*n*_2__′, *y*_*n*_2_+1_,…, *y*_*m*−1_} is rearranged according to the order of small to large, and the new gene **y**′ is obtained.

For gene **t**, a mutation direction **d** is randomly generated in *R* ^*k*^; for the prespecified step size *M*, **t** + *M* · **d** is not in the time window [*a*, *b*]^*k*^; then, set *M* as a random number between 0 and *M*, until it meets the time window [*a*, *b*]^*k*^. If the gene **t** cannot be generated within the specified number of iterations through the above process, then take *M* = 0. Finally, the parent gene **t** is replaced by offspring **t**′ = **t** + *M* · **d**.

### 3.5. Clone Selection

**Figure 3:**
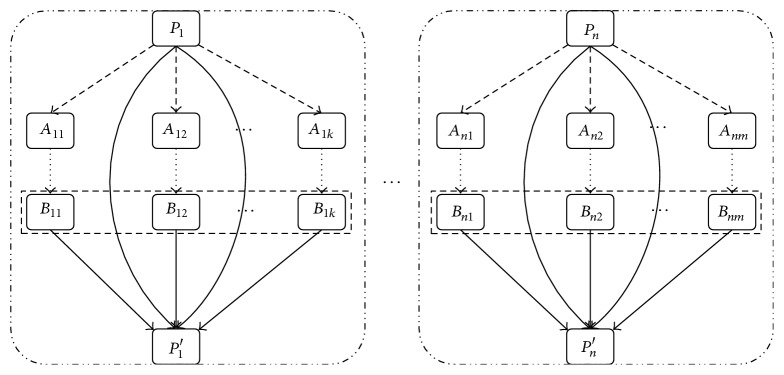
Clonal selection process.

Through crossover and mutation of high frequency, we choose an optimum individual of the highest fitness from parent individuals and offspring as the next generation individuals [21]. The mixture of parent individuals and offspring individuals can avoid degradation of the algorithm. The realization process is shown in Figure 3.

In Figure 3, *P*_1_,…, *P*_*n*_ are parent individuals and *P*_1_ and *P*_*n*_ were copied as *k* and *m*; we perform mutation of high frequency for duplicate individuals; *P*_1_′ and *P*_*n*_′ are offspring individuals after cloning and selection.

## 4. Case Study

**Figure 4:**
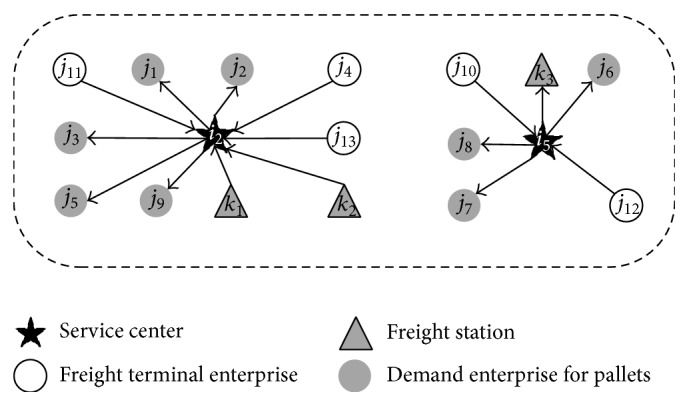
Pallet service area layout.

Now, there are two pallet service centers in the region; they provide pallet delivery and recovery service for each pallet demand point according to the demand in the decision period (as shown in Figure 4). There have direct routes between any two points; the distance from the service center to the demand point and the distance matrix of the demand points are known (as shown in Tables 2 and 3). The number of pallets that will be distributed to the demand point and recovered from the demand points is shown in Table 1. Arrival time of vehicle required by the demand point and the time matrix of vehicle traveling on the road are shown in Tables 4, 5, and 6, and the operation time of vehicles at the point of demand is shown in Table 7. The maximum loading per vehicle is 450 pieces, and the transportation cost of one vehicle is 5 yuan/km. The departure time of the vehicles for each service center is 8:00~14:00 (integral point). Time variables *d*_*v*_^+^ and *d*_*v*_^−^ are all obeying normal distribution (10,1). The stochastic demand *γ*_*v*_ obeys normal distribution (*μ*, *σ*) (as shown in Table 6). The confidence level at which demand points can be serviced in the service window is assumed to be 90%; then, the chance constraint is

(24) $$\begin{array}{c} \mathrm{Pr}\left\{\;T_{v}^{q}\in \left[\;a_{v}-d_{v}^{-},b_{v}+d_{v}^{+}\;\right]\;\right\}\geq 0.9,\;\forall q\in Q,\;\;v\in V. \end{array}$$

The confidence level at which all demand points can be satisfied is 85%; then, the chance constraint is

(25) $$\begin{array}{c} Pr\left\{\;W_{s}^{q}\leq r_{q}\;\right\}\geq 0.85,\;\forall q\in Q,\;\;s\in S. \end{array}$$

**Figure 5:**
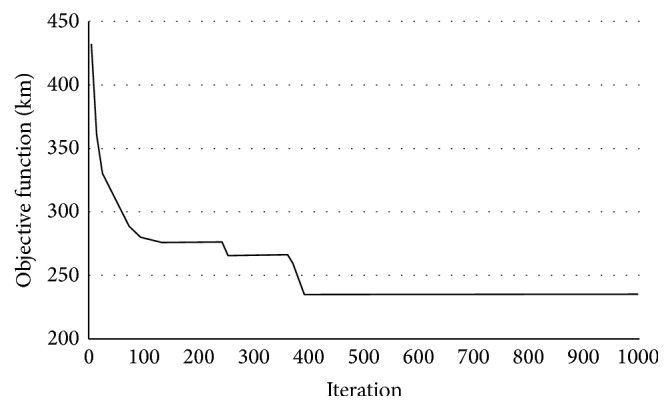
Convergence diagram of hybrid intelligent algorithm for *i*_2_.

**Figure 6:**
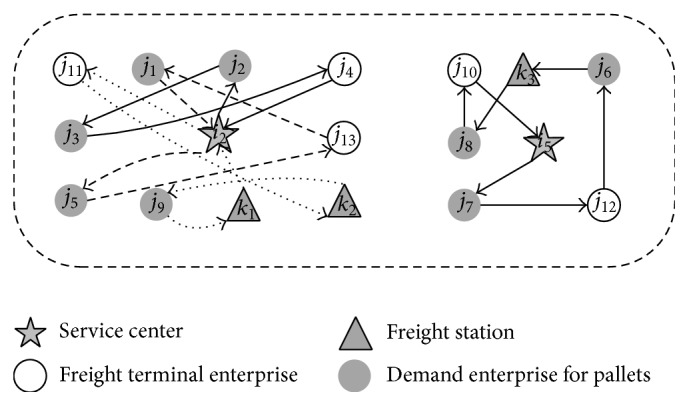
Optimization results of routes under stochastic conditions.

First, we calculate the number of vehicles; the result shows that there need to be 3 vehicles to complete the service for the pallet service center *i*_2_. Then, use hybrid intelligent algorithm to solve the problem (3000 cycle simulations, 1000 clone operations); the algorithm was coded in C++ and run on a Core i5-560M CPU with 2.67 GHz computer. On average, the best solutions were obtained with a population size of 300, and the best result can be found in a very short time.  Figure 5 depicts the convergence diagram of the proposed hybrid intelligent algorithm. The scheme of optimal service routes is as follows (Figure 6):

 The departure time of the first vehicle is 9:00, and walking route is *i*_2_-*j*_11_-*k*_2_-*j*_9_-*k*_1_-*i*_2_.
 The departure time of the second vehicle is 9:00, and walking route is *i*_2_-*j*_2_-*j*_3_-*j*_4_-*i*_2_.
 The departure time of the third vehicle is 10:00, and walking route is *i*_2_-*j*_5_-*j*_13_-*j*_1_-*i*_2_.
 The total length of three vehicles running is 235 km, the total time is 803 minutes, and the total transportation cost is 1175 yuan.

For the pallet service center *i*_5_, it only needs 1 vehicle to complete the service; the scheme of optimal service route is as follows (Figure 6):

 The departure time of vehicle is 14:00, and walking route is *i*_5_-*j*_7_-*j*_12_-*j*_6_-*k*_3_-*j*_8_-*j*_10_-*i*_5_.
 The total length of vehicle running is 106 km, the total time is 329 minutes, and the total transportation cost is 530 yuan.

In addition, if we do not consider the stochastic conditions and time, the scheme of optimal service routes will be as follows.

For the pallet service center *i*_2_,

 the walking route of the first vehicle is *i*_2_-*j*_13_-*j*_9_-*k*_1_-*k*_2_-*i*_2_;
 the walking route of the second vehicle is *i*_2_-*j*_1_-*j*_2_-*j*_4_-*i*_2_;
 the walking route of the third vehicle is *i*_2_-*j*_5_-*j*_3_-*j*_11_-*i*_2_;
 the total length of three vehicles running is 178 km, and the total transportation cost is 890 yuan.

For the pallet service center *i*_5_,

 the walking route of vehicle is *i*_5_-*j*_7_-*j*_8_-*j*_10_-*k*_3_-*j*_6_-*j*_12_-*i*_5_;
 the total length of vehicle running is 106 km, and the total transportation cost is 530 yuan.

**Figure 7:**
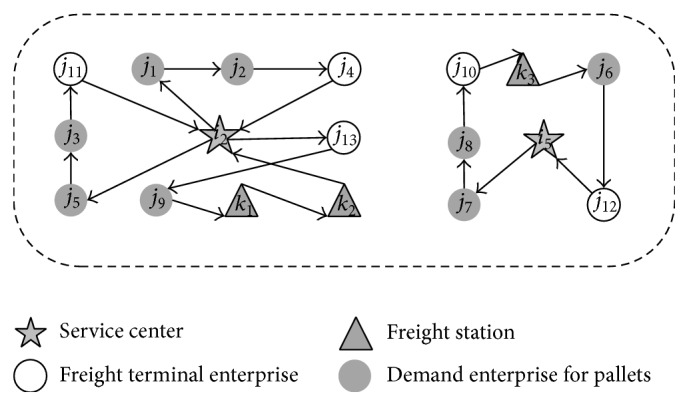
Optimization results without consideration of time and stochastic conditions.

Compared with optimization results without consideration of time and stochastic conditions (Figure 7), the optimization method which considers time and stochastic conditions in the paper costs more time and money, but this can meet the demands of customers. It is more suitable for reality.

## 5. Conclusions

The purpose of route optimization is to find the shortest path of vehicle traveling under the premise of meeting the customers' demand. It has a great impact on sustainability of supply chains. Based on the vehicle routing problem and the Chinese postman problem (CPP), the service route of pallet service center was optimized in this research. Taking full account of various factors that impact the route optimization, a new optimization model with stochastic constraints was formulated and applied to find the best routes of vehicles. Customer demand and transportation time were considered to be stochastic. This assumption adds a more realistic element in the transportation. Because it cannot be solved by optimization software directly, according to the feature of the chance constrained programming model, a novel hybrid intelligent algorithm based on stochastic simulation, neural network, and immune clonal algorithm was proposed for solving the model. Compared with genetic algorithm, the individual affinity of immune clone algorithm includes both the affinity between antibody and antigen (matching degree) and the affinity between antibody and antibody (similarity), so the individual evaluation is more comprehensive and the selection mode for individuals is more reasonable. The validity of the model and algorithm was verified by a case.

However, in actual service, the vehicle loading capacity of pallet service center may be different, and the differences of pallet types have a certain influence on vehicle loading capacity. In addition, the service mode of first delivering and then recycling in the service process might be better. Although the uncertainty of time was considered, it is possible that, due to traffic flow change and traffic accident, the optimal routes may vary. It is necessary to design a real-time route optimizer system. But service route optimization considering the above factors is a complex system engineering problem; it needs to be addressed in future work.

## Figures and Tables

**Table 1:** Delivery and recovery of demand points.

*i*_2_	*j*_1_	*j*_2_	*j*_3_	*j*_4_	*j*_5_	*j*_9_	*j*_11_	*j*_13_	*k*_1_	*k*_2_
Delivery	166	174	269	0	270	182	0	0	0	0
Recovery	0	0	0	61	0	0	134	153	125	108

*i*_5_	*j*_6_	*j*_7_	*j*_8_	*j*_10_	*j*_12_	*k*_3_	—	—	—	—

Delivery	90	83	70	0	0	79	—	—	—	—
Recovery	0	0	0	95	117	0	—	—	—	—

**Table 2:** Distance between service center and the points (km).

	*j*_1_	*j*_2_	*j*_3_	*j*_4_	*j*_5_	*j*_9_	*j*_11_	*j*_13_	*k*_1_	*k*_2_
*i*_2_	5	10	5	3	8	32	5	9	8	15

	*j*_6_	*j*_7_	*j*_8_	*j*_10_	*j*_12_	*k*_3_	—	—	—	—

*i*_5_	0	5	5	8	7	6	—	—	—	—

**Table 3:** Distance matrix between demand points (km).

	*j*_1_	*j*_2_	*j*_3_	*j*_4_	*j*_5_	*j*_9_	*j*_11_	*j*_13_	*k*_1_	*k*_2_	*j*_6_	*j*_7_	*j*_8_	*j*_10_	*j*_12_	*k*_3_
*j*_1_	0	26	22	33	45	50	57	48	34	56	38	42	16	27	31	37
*j*_2_	26	0	9	15	26	18	51	29	41	24	31	43	50	28	19	34
*j*_3_	22	9	0	9	14	17	21	33	20	45	37	21	42	16	31	44
*j*_4_	33	15	9	0	54	15	22	39	43	61	32	18	26	30	19	25
*j*_5_	45	26	14	54	0	28	31	24	37	28	35	14	22	41	29	30
*j*_9_	50	18	17	15	28	0	39	18	20	52	39	47	13	22	36	21
*j*_11_	57	51	21	22	31	39	0	45	39	34	29	24	38	27	42	19
*j*_13_	48	29	33	39	24	18	45	0	29	33	17	20	13	25	47	31
*k*_1_	34	41	20	43	37	20	39	29	0	19	22	37	24	30	16	41
*k*_2_	56	24	45	61	28	52	34	33	19	0	45	23	18	37	22	30
*j*_6_	38	31	37	32	35	39	29	17	22	45	0	21	49	35	17	9
*j*_7_	42	43	21	18	14	47	24	20	37	23	21	0	18	34	29	40
*j*_8_	16	50	42	26	22	13	38	13	24	18	49	18	0	21	35	17
*j*_10_	27	28	16	30	41	22	27	25	30	37	35	34	21	0	36	29
*j*_12_	31	19	31	19	29	36	42	47	16	22	17	29	35	36	0	37
*k*_3_	37	34	44	25	30	21	19	31	41	30	9	40	17	29	37	0

**Table 4:** Travel time matrix from service center to the points (min).

	*j*_1_	*j*_2_	*j*_3_	*j*_4_	*j*_5_	*j*_9_	*j*_11_	*j*_13_	*k*_1_	*k*_2_
*i*_2_	10	20	10	6	16	64	10	18	17	31

	*j*_6_	*j*_7_	*j*_8_	*j*_10_	*j*_12_	*k*_3_	—	—	—	—

*i*_5_	0	11	10	16	15	12	—	—	—	—

**Table 5:** Theory travel time matrix between the demand points (min).

	*j*_1_	*j*_2_	*j*_3_	*j*_4_	*j*_5_	*j*_9_	*j*_11_	*j*_13_	*k*_1_	*k*_2_	*j*_6_	*j*_7_	*j*_8_	*j*_10_	*j*_12_	*k*_3_
*j*_1_	0	52	44	60	90	100	114	96	68	112	76	84	32	54	62	74
*j*_2_	52	0	18	30	52	36	102	58	82	48	62	86	100	56	38	68
*j*_3_	44	18	0	18	28	34	42	66	40	90	74	42	84	32	62	88
*j*_4_	66	30	18	0	108	30	44	78	86	122	64	36	52	60	38	50
*j*_5_	90	52	28	108	0	56	62	48	74	56	70	28	44	82	58	60
*j*_9_	100	36	34	30	56	0	78	36	40	104	78	94	26	44	72	42
*j*_11_	114	102	42	44	62	78	0	90	78	68	58	48	76	54	84	38
*j*_13_	96	58	66	78	48	36	90	0	58	66	34	40	26	50	94	62
*k*_1_	68	82	40	86	74	40	78	58	0	38	44	74	48	60	32	82
*k*_2_	112	48	90	122	56	104	68	66	38	0	90	46	36	74	44	60
*j*_6_	76	62	74	64	70	78	58	34	44	90	0	42	98	70	34	18
*j*_7_	84	86	42	36	28	94	48	40	74	46	42	0	36	68	58	80
*j*_8_	32	100	84	52	44	26	76	26	48	36	98	36	0	42	70	34
*j*_10_	54	56	32	60	82	44	54	50	60	74	70	68	42	0	72	58
*j*_12_	62	38	62	38	58	72	84	94	32	44	34	58	70	72	0	74
*k*_3_	74	68	88	50	60	42	38	62	82	60	18	80	34	58	74	0

**Table 6:** Arrival time window and random demand for demand points.

Demand points	Time windows	*μ*	*σ*
*j*_1_	[13:00, 15:20]	5	3
*j*_2_	[8:10, 12:20]	4	1
*j*_3_	[10:00, 12:00]	2	1
*j*_4_	[10:15, 12:00]	—	—
*j*_5_	[9:20, 11:00]	3	1
*j*_9_	[10:25, 13:00]	4	2
*j*_11_	[8:30, 11:30]	—	—
*j*_13_	[9:00, 13:00]	—	—
*k*_1_	[13:00, 14:30]	—	—
*k*_2_	[10:00, 12:20]	—	—
*j*_6_	[14:25, 16:40]	2	1
*j*_7_	[13:30, 16:10]	6	3
*j*_8_	[16:00, 18:00]	4	2
*j*_10_	[17:40, 19:00]	—	—
*j*_12_	[13:45, 16:35]	—	—
*k*_3_	[14:30, 17:00]	4	2

**Table 7:** Operation time of vehicle at the demand points (min).

*i*_2_	*j*_1_	*j*_2_	*j*_3_	*j*_4_	*j*_5_	*j*_9_	*j*_11_	*j*_13_	*k*_1_	*k*_2_
Operation time	32	40	55	15	56	42	25	28	20	19

*i*_5_	*j*_6_	*j*_7_	*j*_8_	*j*_10_	*j*_12_	*k*_3_	—	—	—	—

Operation time	20	18	17	22	20	19	—	—	—	—

## References

[1] Ruan Q.-F. (2012). *Research on the Vehicle Routing Problem with Pickups and Deliveries under Handling Policies*.

[2] Toth P., Vigo D. (2002). *The Vehicle Routing Problem: SIAM Monographs on Discrete Mathematics and Applications*.

[3] Hoff A., Gribkovskaia I., Laporte G., Løkketangen A. (2009). Lasso solution strategies for the vehicle routing problem with pickups and deliveries. *European Journal of Operational Research*.

[4] Psaraftis H. N. (1988). *Dynamic Vehicle Routing Problems*.

[5] Psaraftis H. N. (1995). Dynamic vehicle routing: status and prospects. *Annals of Operations Research*.

[6] Larsen A. (2001). *The Dynamic Vehicle Routing Problem*.

[7] Brotcorne L., Laporte G., Semet F. (2003). Ambulance location and relocation models. *European Journal of Operational Research*.

[8] Taniguchi E., Shimamoto H. (2004). Intelligent transportation system based dynamic vehicle routing and scheduling with variable travel times. *Transportation Research Part C: Emerging Technologies*.

[9] Fleischmann B., Gnutzmann S., Sandvoss E. (2004). Dynamic vehicle routing based on online traffic information. *Transportation Science*.

[10] Melachrinoudis E., Ilhan A. B., Min H. (2007). A dial-a-ride problem for client transportation in a health-care organization. *Computers & Operations Research*.

[11] Khouadjia M. R., Sarasola B., Alba E., Jourdan L., Talbi E.-G. (2012). A comparative study between dynamic adapted PSO and VNS for the vehicle routing problem with dynamic requests. *Applied Soft Computing*.

[12] Ferrucci F., Bock S., Gendreau M. (2013). A pro-active real-time control approach for dynamic vehicle routing problems dealing with the delivery of urgent goods. *European Journal of Operational Research*.

[13] Albareda-Sambola M., Fernández E., Laporte G. (2014). The dynamic multiperiod vehicle routing problem with probabilistic information. *Computers & Operations Research*.

[14] Ghannadpour S. F., Noori S., Tavakkoli-Moghaddam R., Ghoseiri K. (2014). A multi-objective dynamic vehicle routing problem with fuzzy time windows: model, solution and application. *Applied Soft Computing*.

[15] Guan M.-G. (1960). Operation method on parity point. *Acta Mathematica Sinica*.

[16] Edmonds J. (1965). The Chinese postman problem. *Operation Research*.

[17] Wang S.-H. (1995). Many postmen Chinese postmen problems. *Journal of University of Science and Technology of China*.

[18] Fei R., Cui D.-W., Wang Z.-M., Liang K. (2006). Motion planning algorithms for certain many postmen Chinese postmen problems. *Journal of Zhengzhou University: Natural Science Edition*.

[19] Zhu S.-D. (2006). *Discussion on Vehicle Collocating and Each Route of Layer Logistics System [EB/OL]*.

[20] Liu B.-D., Zhao R.-Q., Wang G. (2003). *Uncertain Programming with Applications*.

[21] Li L., Li H.-Q., Xie S.-L., Li X.-Y. (2008). Immune particle swarm optimization algorithms based on clone selection. *Computer Science*.

